# Comparison of hybrid volumetric modulated arc therapy (VMAT) technique and double arc VMAT technique in the treatment of prostate cancer

**DOI:** 10.1515/raon-2015-0018

**Published:** 2015-08-21

**Authors:** Christopher Amaloo, Daryl P. Nazareth, Lalith K. Kumaraswamy

**Affiliations:** 1 Department of Radiation Medicine, Roswell Park Cancer Institute, Buffalo, NY, USA; 2 Department of Radiation Medicine, Roswell Park Cancer Institute and Department of Biophysics and Physiology, University at Buffalo, Buffalo, USA; 3 Department of Radiation Medicine and Department of Cell Stress Biology, Roswell Park Cancer Institute, Buffalo, USA

**Keywords:** VMAT, hybrid, prostate, planning

## Abstract

**Background:**

Volumetric modulated arc therapy (VMAT) has quickly become accepted as standard of care for the treatment of prostate cancer based on studies showing it is able to provide faster delivery with adequate target coverage and reduced monitor units while maintaining organ at risk (OAR) sparing. This study aims to demonstrate the potential to increase dose conformality with increased planner control and OAR sparing using a hybrid treatment technique compared to VMAT.

**Methods:**

Eleven patients having been previously treated for prostate cancer with VMAT techniques were replanned with a hybrid technique on Varian Treatment Planning System. Multiple static IMRT fields (2 to 3) were planned initially based on critical OAR to reduce dose but provide some planning treatment volume (PTV) coverage. This was used as a base dose plan to provide 30–35% coverage for a single arc VMAT plan.

**Results:**

The clinical VMAT plan was used as a control for the purposes of comparison. Average of all OAR sparing between the hybrid technique and VMAT showed the hybrid plan delivering less dose in almost all cases except for V80 of the bladder and maximum dose to right femoral head. PTV coverage was superior with the VMAT technique. Monitor unit differences varied, with the hybrid plan able to deliver fewer units 37% of the time, similar results 18% of the time, and higher units 45% of the time. On average, the hybrid plan delivered 10% more monitor units.

**Conclusions:**

The hybrid plan can be delivered in a single gantry rotation combining aspects of VMAT with regions of dynamic intensity modulated radiation therapy (IMRT) within the treatment arc.

## Introduction

Prostate cancer is one of the most prevalent malignant diseases that occur among men with a new case diagnosed every 2.2 minutes, affecting 1 in 6 men in their lifetime.^1^,^2^ Traditionally, radiotherapy has been a vital part of the treatment of prostate cancer with three-dimensional conformal radiotherapy (3D-CRT) as the historical standard. Data from dose escalation studies suggests an association between increased dose and an improvement in prostate cancer control^3^ with an increased efficacy in prostate-specific antigen (PSA) control at the cost of increased toxicity.^4^,^5^ By utilizing techniques such as intensity modulated radiation therapy (IMRT) or volumetric modulated arc therapy (VMAT) with image guided radiotherapy (IGRT), the amount of normal tissue treated can be reduced and thus limit this increased toxicity.^6^,^7^ Due to these improved outcomes, IMRT and VMAT techniques are becoming the new standard for curative external beam radiation therapy.^7^–^9^ Given the widespread and prolific nature of the disease, it has proven to be a vital site for the validation of new treatment modalities. Previous studies have shown that VMAT offers reduced monitor units (MU) and delivery time in comparison to IMRT^10^–^15^, at the cost of low dose spillage and potentially reduced conformation of dose for all treatment sites.

RapidArc® is a form of VMAT that delivers intensity modulated radiation arcs by simultaneously changing gantry speed, multileaf collimator (MLC) position, and dose rate.^11^ While this technique offers increased dose conformality and sparing of organs at risk (OAR) compared to 3D-CRT^16^, one drawback of such a technique is the spread of low dose to the surrounding normal tissue.^11^–^17^ In the treatment of prostate cancer, the spread of such a large low dose region can lead to issues with the intestinal tract, causing such secondary issues as diarrhea, intestinal strictures, and incontinence.^9^,^18^ In order to reduce the low dose volume, and achieve better control to the respective OARs, a hybrid technique was developed similar in nature to Chan *et al.* with the use of dynamic IMRT in place of 3D conformal fields.^19^ Our hybrid technique features a pair of non-opposing dynamic IMRT fields where the beam axis covers the planning treatment volume (PTV) while minimizing the overlap with the OARs. It contributed approximately 1/3 of the total dose to the targets, with the remaining 2/3 of the dose coming from a single overlaying VMAT arc.

The aim of this study is to retrospectively compare the dosimetric parameters of this hybrid treatment technique combining the use of dynamic IMRT fields supplementing a single modulated arc pass to the standard VMAT plan containing two full arcs frequently utilized clinically at our institute for the treatment of prostate cancer.

## Methods

Eleven patients, previously treated at our institute for prostate cancer, were chosen for this study. A variety of treatment plans were chosen to include a combination of patient size, target size, and compromised critical OARs that require special consideration. Patient age ranged from 60 to 81 with a mean age of 68. Patients had PSA scores ranging from of 4.4 to 24.4, with Gleason scores from 6 to 9 (Table 1).

All patients were treated clinically with a VMAT technique, utilizing two arcs to achieve a conformal dose to the target structure. These patients were then retrospectively re-planned with a hybrid technique on Varian Eclipse Treatment Planning Software (TPS) (Varian Medical Systems, Palo Alto, CA) Version 10.0. The hybrid technique consisted of dual dynamic IMRT fields with geometry designed to limit OAR dose but provide some PTV coverage. Dose was calculated and subsequently used as a base dose plan, providing initial coverage for a single overlay volume modulated arc.

Patients were simulated with a GE Lightspeed computed tomography simulator in the supine position on a flat tabletop. A custom formed vacloc bag was utilized to ensure consistent setup and stabilization. The bladder at time of simulation was filled to a degree that was maintainable and reproducible for daily treatment. CT slices were acquired at a thickness of 2.5 mm covering a region from above the iliac crests superiorly to below the perineum inferiorly.

A physician contoured the gross tumor volume (GTV) to include all known disease, as defined by the planning CT, encompassing the entirety of the prostate gland. A urethrogram was used in planning to aid in delineation of the inferior border of the prostate to include a volume 5 mm superior to the tip of the dye. The clinical treatment volume (CTV) is the GTV and areas of microscopic disease extension including the proximal 1 cm of the seminal vesicles. The PTV included a 1 cm radial expansion of the CTV in all directions, except a posterior margin of 6 mm, to allow for treatment set up variation as well as internal motion of organs. The confidence in the reduced size of the posterior margin is due, in part, to IGRT techniques of weekly CBCT and daily kV orthogonal matching to imbedded radiopaque fiducial prostate seed markers.

OARs contoured on the treatment planning CT include the left and right femoral heads to the level of the ischial tuberosity, the bladder, and the rectum from the superior rectosigmoid flexure to the inferior level of the ischial tuberosities. Additionally, an external body structure was also contoured as a normal tissue for the purposes of dose volume histogram (DVH) analysis. All structures contoured exist as solid organs in their entirety.

The prescription dose was 1.8 Gy per fraction for 44 fractions (79.2 Gy total dose) to cover 95% of the PTV, with the maximum dose in the PTV no more than 107% of the prescription dose.

### VMAT planning

All treatment planning was performed on Varian Eclipse TPS 10.0. The original VMAT plans were copied and dose was calculated based on the previously generated arc parameters with the following criteria: The treatment isocenter was placed at the center of the PTV. Two full arcs were planned using the Eclipse Arc Geometry Tool, with the initial arc rotating clockwise from 181° to 179°, a collimator rotation of 30° and the second arc rotating counter clockwise from 179° back to 181° with a collimator rotation of 320°.

**FIGURE 1.**
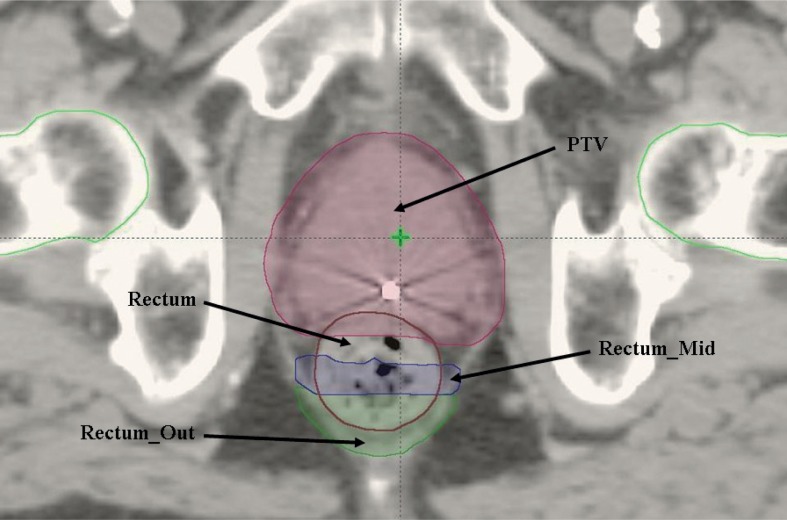
Diagram showing additional structures to control rectal dose.

Additional structures created for planning purposes only included a radial expansion of 1 mm on the PTV to enhance dose coverage and fall off. Regions of overlap between treatment volumes and OARs were contoured to control dose effectively within these areas. Additionally, to better control dose to the rectum, two additional structures were contoured as illustrated in Figure 1. Rectum_Out was a radial expansion of 5 mm on the rectum minus the overlap contour, subsequently cropped out of the overlap area with an additional 1 cm margin. Rectum_Mid was a radial expansion of 5 mm on the rectum minus the overlap contour, subsequently cropped out of both the overlap area with a 3 mm margin and Rectum_Out. Finally, support structures were added to account for the treatment couch in the path of the arcs.

Within the VMAT optimizer, using calculation model algorithm PRO_10028, upper and lower dose constraints were set on all tumor and treatment volumes and expansions thereof. Coverage of targets thus defined received topmost priority, with OARs receiving lesser priority in the order of rectum, bladder, and finally, bilateral femoral head and necks. The Normal Tissue Objective was utilized, with a priority value matching that of the target coverage, with automatic tissue sparing selected.

Dose was calculated for an intermediate optimization, and final dose calculated after the VMAT optimizer was run a second time to completion. A normalization point was selected such that 100% of the prescribed dose would be delivered to 95% the PTV.

### Hybrid planning

The hybrid planning technique was comprised of two dynamic IMRT fields with a single overlying volume modulated arc delivered to the same iso-center as in the VMAT plans. Beam arrangement of the dynamic fields was chosen such that the majority of the PTV on the central axis received coverage while minimizing direct OAR exposure within the fields. Directly opposed fields were avoided, and in general, left and right anterior oblique fields gave the best geometry.

The two dynamic IMRT fields provided approximately 1/3 of the total dose, with the remaining dose supplied by the overlying arc. The IMRT fields served as a base dose for the VMAT optimizer, with the same structures and constraints as utilized for the VMAT planning process.

Again, dose was calculated for an intermediate optimization, and final dose calculated with the VMAT optimizer run to completion on a second pass. A normalization point was chosen to achieve the same coverage as in the VMAT plan.

Isodose lines were analyzed and, if possible, the IMRT fluences were adjusted manually to increase target coverage or reduce hot spots.

### Analysis

For each case, the two competing treatment plans were compared on the basis of several criteria. For target coverage, PTV min (D2), max (D98), and mean, as recommended by ICRU 83^20^ for dose reporting, as a percentage of prescribed dose were cross referenced against a conformality index (CI) as defined below. For OAR analysis, the data was examined based on the specific organ tolerances as per tables in RTOG 0815. For the rectum, the DVH points of D15, D25, D35, and D50 as well as the V80, V75, V70 and V65 were examined. For the bladder, the DVH points of interest were again D15, D25, D35, and D50 as well as V75, V70, V65, and V60. For the bilateral femora both the max and mean values were compared. To gauge low dose to the body, the body V5 and V10 was used as a point of comparison, as well as a calculation for integral dose (ID) as defined below. Finally, a monitor unit comparison was made between the hybrid and VMAT plan as an indicator of modulation.

[1] CI=VRx/V_PTV

Where V_Rx_ is the volume in cc receiving the prescription dose, and V_PTV_ is the PTV volume in cc.

[2] $$\mathrm{ID}=Vp\bar{D}$$

Where V, p, and *D̄* are respectively the volume, density of the organ, and mean organ dose.

## Results

**FIGURE 2.**
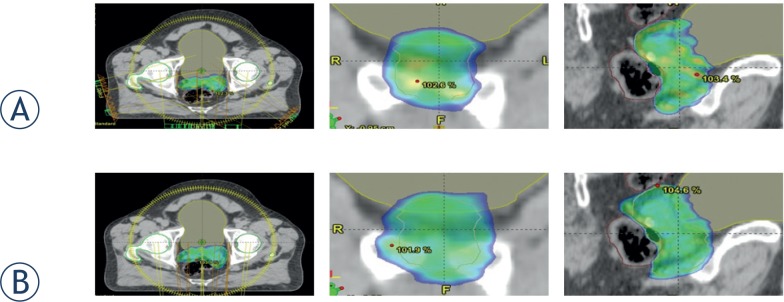
Dose distribution for typical hybrid **(A)** and 2 arc volumetric modulated arc therapy (VMAT) **(B)**. plans with color wash of 95% of the prescription dose.

**FIGURE 3.**
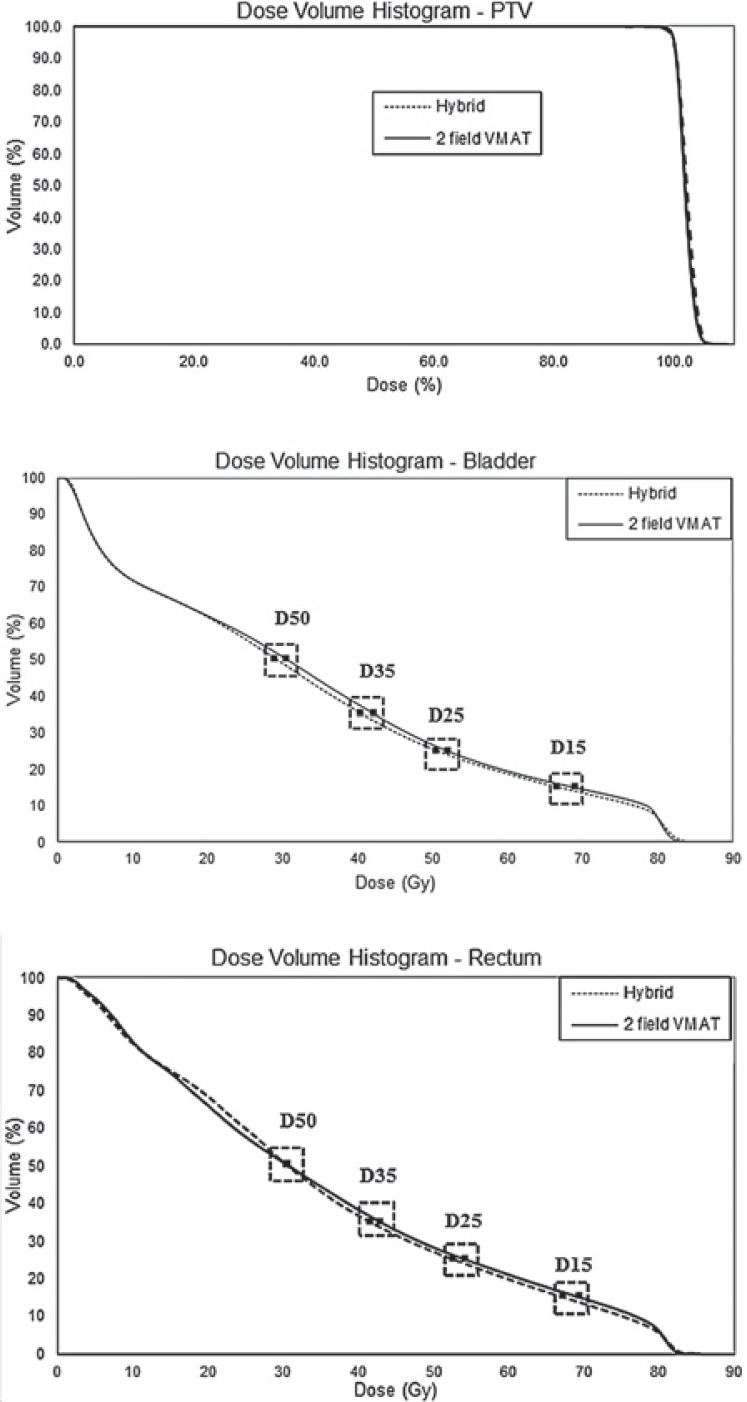
Dose volume histogram (DVH) comparison of some planning treatment volume (PTV), rectum, and bladder between the hybrid technique and the two field volumetric modulated arc therapy (VMAT) technique.

Dose color wash at 95% of the prescription dose is shown for the hybrid treatment and for the double arc VMAT comparison in Figure 2. The breakdown of target coverage with CI, PTV minimum, maximum, and mean along with MU delivered and ID are shown in Table 2. The OAR study parameters for the two techniques are tabulated in Table 3 with corresponding differences. The average DVHs for the PTV and bladder and rectum are shown in Figure 3.

### Target coverage

The hybrid plan had better conformality compared to the double arc VMAT plan with a relative improvement of 5.5% in CI. All of the plans were considered acceptable with 95% of the PTV volume receiving 100% of the prescribed dose, but the VMAT plan achieved better dose homogeneity with an increase in 1% to PTV minimum and a reduction of 1% in PTV maximum (Table 2).

#### Organs at risk

Critical structure dose constraints followed RTOG protocol 0815 such that volumes of 15%, 25%, 35%, and 50% shall receive doses less than 80, 75, 70 and 65 Gy for the bladder and 75, 70, 65, 60 Gy for the rectum, respectively. The clinical VMAT plan served as control for the purposes of comparison. On average, the hybrid technique provided greater OAR sparing for both the rectum and bladder at all volumes and doses of interest (Table 3). For the volume constraints, the p-value of each difference is provided. A low p-value indicates that the VMAT and hybrid values are statistically significantly different.

### Monitor units, integral dose, low dose spillage

Monitor unit differences varied, with the hybrid plan able to deliver fewer MU 45% of the time. Integral dose was slightly lower with the hybrid plan. For low doses of radiation to the whole body, the hybrid plan fared slightly better than the double arc VMAT plans, with a reduction in V5 of 0.8%, and a reduction in V10 of 1.9% on average (Table 2, 3).

## Discussion

Previous studies comparing VMAT to IMRT for prostate treatment have highlighted the fact that VMAT delivery is more efficient than that of IMRT.^16^, ^21^–^33^ However, all of these studies have mixed dosimetric results. Some studies have shown better sparing of OARs with IMRT^23^, while some have shown equivalent sparing of OARs.^26^,^33^ For example, the study by Ishii *et al.*^33^ showed that PTV coverage was similar between RapidArc, 7 field IMRT, and 9 field IMRT. For the rectum D_mean_, V_65Gy_ and V_45Gy_ and bladder V_45Gy_, their results indicated that 9 field IMRT plans had significantly lower values than the RapidArc and 7 field IMRT. Our aim was to develop a technique that would further reduce the delivery time and maintain the same level of dosimetric outcome as the conventional RapidArc delivery.

The hybrid technique enables the planner to more easily control dose to critical OARs. The use of dynamic IMRT allows for additional input into the TPS and serves as a portal for the planner to more directly control dose distributions. The combination of directly changing fluencies for the IMRT portion of delivery, as well as a choice of beam geometry allows the planner to better control where dose falls. Greater OAR sparing was seen in the hybrid technique for both bladder and rectum points across the range of interest.

The hybrid technique provides greater conformality compared to the standard VMAT. As IGRT localization improves, this allows for reduced OAR volumes exposed to escalated doses, potentially reducing toxicity to normal tissues. This not only creates the opportunity for reduced patient complications, but also room for dose escalation leading to potential increased local control. One way of measuring the dose-modulation potential of a plan delivery modality is to consider the number of control points it allows. The control points are created by the TPS software and contained in a treatment plan’s DICOM file. Each control point specifies the state of the linac at a given instant of treatment (*i.e.*, jaw settings, MLC positions, MUs, gantry angle and rotation speed, etc.). An IMRT plan and full-arc VMAT plan contain 320 and 178 control points, respectively. Therefore, a hybrid plan contains 2×320+178 = 818 control points, while double-arc VMAT plan contains 178×2 = 356 control points. Thus, the hybrid plan provides better dose modulation and control of dose fall off around the PTV.

With Varian Eclipse treatment planning, the hybrid plan was created utilizing separate optimizers from the constituent VMAT and IMRT portions. With this method of planning, the IMRT base dose plan is unaware of the subsequent VMAT arc and therefore cannot effectively yield homogeneous coverage to the PTV. This inhomogeneous base dose presents an additional restriction on the VMAT optimizer to achieve constraints as seen by the decrease in PTV homogeneity of 2% with the hybrid technique.

A typical prostate VMAT field has a beam on time of 1.2 minutes (72 seconds) during treatment. Since most of our prostate VMAT plans require two arcs to achieve a homogeneous dose distribution to the target, the combined beam on time is about 2.4 minutes (144 seconds). The hybrid plan, on the other hand, can be delivered in one gantry sweep consisting of two dynamic IMRT fields with a single overlying VMAT field. A typical dynamic IMRT field from this technique has a beam on time of 0.3 minute. Therefore, the hybrid technique would have a typical total beam on time of 1.2 min + 0.3 min + 0.3 min = 1.8 min, or 108 seconds, which is approximately 0.5 min lower than the typical prostate VMAT delivery in our clinic. Thus, this reduction in time would reduce the chance of intra-fraction motion and increasing patient comfort. Delivery of the treatment in one gantry rotation can also reduce machine wear and potential down-time for machine maintenance.

Total MUs for both techniques are similar, but with a lower ID with the hybrid technique, as well as reduced volume receiving 5 Gy and 10 Gy overall. This is critical due to the radiosensitivity of the OAR’s, particularly the rectum and bladder.

## Conclusions

Dosimetrically the hybrid and double arc VMAT plans are similar, with the hybrid plan achieving better constraints on the OARs without significantly higher MUs.

Clinically, the hybrid plan offered slightly poorer homogeneity compared with the VMAT plans, yielding a greater shoulder on the PTV coverage. This issue is due to the nature of the two disparate optimizers attempting to achieve overall dose homogeneity. Changing the nature of the optimization to a single overall algorithm would correct the difficulty in achieving completely uniform coverage.

The hybrid treatment delivered in a single gantry rotation with short pauses for small IMRT corrections could potentially reduce treatment time and increase target localization compared to multiple arc VMAT, while providing superior sparing of critical OARs. It not only allows for increased conformality of dose over VMAT while maintaining reduced treatment times over full IMRT, but also provides more planner control in the area of low dose spillage.

## Figures and Tables

**TABLE 1.** Patient characteristics

**Pt number**	**Staging**	**Gleason score**	**PSA**	**Age**
1	T1c	4+3=7/10	6.0	70
2	T1c	3+3=6/10	4.4	67
3	T1c	3+4=7/10	9.9	61
4	T2a	4+5=9/10	24.4	81
5	T2c	3+3=6/10	10.8	67
6	T2a	5+4=9/10	9.5	74
7	T1c	3+4=7/10	6.1	70
8	T1c	4+3=7/10	8.8	60
9	T1c/T2a	4+3=7/10	4.8	69
10	T1c	4+3=7/10	6.8	63
11	T2b/T3a	4+5=9/10	14.2	63

PSA = prostate-specific antigen; Pt = patient

**TABLE 2.** Planning treatment volume (PTV) coverage, monitor units, and integral dose for delivery of plans

**Pt number**	**Modality**	**CI**	**PTV-min**	**PTV-max**	**PTV-mean**	**MU**	**Integral dose**
1	Hybrid	0.98	92.8%	108.5%	103.0%	686	308.1
	VMAT	1.08	95.5%	108.1%	101.4%	867	303.7
2	Hybrid	0.99	91.3%	107.1%	102.3%	817	186.5
	VMAT	1.15	97.5%	107.6%	102.0%	697	198.8
3	Hybrid	1.00	94.4%	107.6%	102.3%	616	255.8
	VMAT	1.05	96.2%	106.2%	101.3%	592	255.3
4	Hybrid	1.05	95.0%	107.7%	102.5%	743	200.3
	VMAT	1.08	97.5%	109.4%	101.5%	574	196.3
5	Hybrid	1.07	94.9%	106.6%	102.4%	521	232.0
	VMAT	1.12	96.9%	106.9%	101.3%	796	233.1
6	Hybrid	1.15	96.8%	111.0%	101.6%	600	216.3
	VMAT	1.32	91.6%	109.8%	102.0%	602	208.6
7	Hybrid	0.97	94.2%	107.1%	102.8%	542	201.3
	VMAT	0.97	94.7%	106.1%	102.3%	487	201.8
8	Hybrid	1.03	95.2%	110.0%	102.6%	729	133.5
	VMAT	1.03	94.8%	107.4%	101.7%	598	132.2
9	Hybrid	1.09	95.3%	109.7%	102.3%	786	254.7
	VMAT	1.17	97.4%	106.2%	101.6%	622	263.5
10	Hybrid	1.13	96.2%	109.3%	101.8%	603	178.5
	VMAT	1.03	92.6%	107.3%	102.2%	610	175.6
11	Hybrid	1.00	96.0%	106.6%	102.1%	521	165.6
	VMAT	1.14	98.1%	106.0%	101.7%	587	175.5
AVE	Hybrid	1.04	94.74%	108.29%	102.34%	651.27	212.05
	VMAT	1.10	95.71%	107.36%	101.73%	639.27	213.13

AVE = average; CI = confidence interval; MU = monitor units; Pt = patient; VMAT = volumetric modulated arc therapy

**TABLE 3.** Organ-at-Risk constraints for bladder, rectum, femora and body

**Dose Constraints**												

	**Bladder**				**Rectum**				**Left F. Head**		**Right F. Head**	
	***D50***	***D35***	***D25***	***D15***	***D50***	***D35***	***D25***	***D15***	**Max**	**Mean**	**Max**	**Mean**
**VMAT (Gy)**	30.66	42.35	52.12	69.11	30.75	42.98	54.31	69.50	38.49	17.31	37.13	17.18
**HYBRID (Gy)**	29.10	40.56	50.62	66.72	30.66	41.69	52.83	67.34	44.86	15.41	46.02	18.25
**DIFF (Gy)**	1.56	1.79	1.50	2.40	0.09	1.28	1.48	2.16	−6.37	1.90	−8.89	−1.07

**Volume Constraints**											

	**Bladder**				**Rectum**					**Body**	
	***V65***	***V70***	***V75***	***V80***	***V50***	***V60***	***V65***	***V70***	***V75***	***V5***	***V10***
**VMAT (%)**	16.92	14.60	12.28	6.73	28.08	21.15	17.97	14.92	11.57	25.34	20.40
**HYBRID (%)**	15.88	13.40	10.96	6.43	26.24	19.47	16.10	12.93	9.69	24.51	18.51
**DIFF (P-Val)**	1.04 (0.36)	1.20 (0.32)	1.32 (0.27)	0.30 (0.39)	1.84 (0.31)	1.68 (0.25)	1.87 (0.17)	1.99 (0.09)	1.88 (0.06)	0.84	1.89

DIFF = difference; f. = femur; VMAT = volumetric modulated arc therapy
